# Riding the knowledge translation roundabout: lessons learned from the Canadian Institutes of Health Research Summer Institute in knowledge translation

**DOI:** 10.1186/1748-5908-4-33

**Published:** 2009-06-12

**Authors:** Michelle E Kho, Elizabeth A Estey, Ryan T DeForge, Leanne Mak, Brandi L Bell

**Affiliations:** 1 Department of Clinical Epidemiology and Biostatistics, McMaster University, Hamilton, ON, Canada; 2 Centre for Aboriginal Health Research, University of Victoria, Victoria, BC, Canada; 3 Li Ka Shing Knowledge Institute, St. Michael's Hospital, Toronto, ON, Canada; 4 Department of Health and Rehabilitation Sciences, The University of Western Ontario, London, ON, Canada; 5 Department of Psychology, University of Manitoba, Winnipeg, MB, Canada; 6 Comprehensive School of Health Research, University of Prince Edward Island, Charlottetown, PEI, Canada

## Abstract

**Background:**

Funding the education and training of the next generation of health researchers is a key mandate of the Canadian Institutes of Health Research (CIHR) knowledge translation (KT) portfolio. The field of KT is growing daily; thus, the training and development of a new generation of KT researchers is essential.

**Methods:**

Using curriculum documents, participant evaluations, and self-reflection, this paper describes a unique Summer Institute hosted by the CIHR in Cornwall, Ontario, Canada. We outline the key aspects of a successful training initiative that could inform organizations and agencies worldwide with an interest in or who have a mandate for KT.

**Results:**

This work provides potential funders, faculty, and students with an inside look into the purpose, process, and outcomes of such training initiatives.

**Conclusion:**

National and international KT organizations, research institutions, and funding agencies are encouraged to consider replicating the training model employed here, as investment into KT personnel will foster the advancement of the field within and beyond local borders.

'To the individual who devotes his/her life to science, nothing can give more happiness than when the results immediately find practical application. There are not two sciences. There is science and the application of science, and these two are linked as the fruit is to the tree.' – Louis Pasteur, 1871 (from presentation by Ian Graham, 2008 CIHR Knowledge Translation Summer Institute)

## Introduction

Knowledge translation (KT) is a young field that is grappling with its definition, terminology, and methodologies [1,2]. At the most basic level, however, KT is about putting knowledge into action. In this paper, we use the Canadian Institutes of Health Research (CIHR) definition of KT: *'*a dynamic and iterative process that includes synthesis, dissemination, exchange, and ethically sound application of knowledge to improve the health of Canadians, provide more effective health services and products, and strengthen the healthcare system' [3]. With a legal mandate for KT, the CIHR has made significant contributions that are recognized both nationally and internationally [4]. Funding education and training of the next generation of Canadian health researchers in KT is an important part of the CIHR's KT portfolio; formal opportunities to develop and train new KT researchers and experts are needed by healthcare systems to ensure that a mandate for KT is sustained within the research and decision-making communities [3].

One example of a training initiative is the CIHR's Innovation in Knowledge Translation Research and Knowledge Translation Summer Institute (KTSI), which occurred from 22 to 25 June 2008 in Cornwall, Ontario, Canada. This intensive, four-day strategic capacity-building institute was funded by the CIHR's Institutes of Health Services and Policy Research (IHSPR), Population and Public Health (IPPH), and the Knowledge Synthesis and Exchange Branch. Dr. Jeremy Grimshaw of the CIHR funded KT-ICEBERG (Improving Clinical Effectiveness through Behavioural Research Group) [5], and the Clinical Epidemiology Program of the Ottawa Hospital Research Institute (OHRI) was the host. Through faculty engagement and a variety of different teaching methods, 30 Canadian trainees actively learned about the science of KT.

The KTSI had four specific aims, focusing on health services and policy or population and public health areas: explore the challenges of planning and carrying out KT research and KT involving and/or engaging different stakeholder groups; increase the understanding of concepts, methods, and theories relevant to KT research, including learning about the concepts that underlie the evidentiary base for effective KT targeting different decision-making groups, and investigating the contribution of different disciplinary and methodological approaches.

### Explore ethical issues associated with KT research and KT

In contrast to a meeting report written by course tutors, we are five of the meeting participants (brought together through small group work during the KTSI) and present an end-user perspective of this training initiative. Using curriculum documents, participant evaluations, and self-reflection, we use this paper to share the teaching model of the KTSI curriculum, document our experiences, and present some of the key lessons learned. We believe that the KTSI model is a helpful starting point to inform other funding agencies or research groups who wish to develop new researchers and experts in the KT field.

### The KTSI structure and curriculum

#### The application process

Over 150 trainees applied to fill the 30 spots available for the KTSI through a competitive process. The CIHR encouraged applications from different disciplines; however, applicants must have had research interests in KT research or in integrating KT into their research. The selection committee assessed each application based on the candidate's academic status (five points, preference to PhD students or postdoctoral fellows), research awards held (five points, preference to those holding research awards) and written responses to three essay questions (40 points; Appendix 1 outlines the KTSI questions applicants completed). Two independent reviewers assessed each application using a block design so that each reviewer was also paired with every other reviewer for at least five applications. The *a priori *cutoff score for inclusion was 80% (40 of 50 points).

Almost all successful applicants (97%) were enrolled in doctoral studies or held postdoctoral fellowships focused on KT, and 80% held CIHR awards. Participants represented 16 different Canadian institutions, and a variety of faculties and departments, including communications, engineering, health promotion, and political science. Additional file 1 outlines the research projects and interests of the authors (responses to Appendix 1, question one).

#### Curriculum

Twelve faculty with KT expertise representing Canada, the United States, and the United Kingdom, shared their knowledge and experience with trainees. Faculty purposefully designed the curriculum to expose participants to basic research methodology in KT, varied areas of KT research and applications of KT targeted towards different stakeholder groups (e.g. public, clinicians, and policy makers), international perspectives of KT, and ethics of KT research. The KTSI included plenary presentations, concurrent sessions aimed at skill building in methods and/or research techniques and interactive case studies. A small group activity focused on developing, implementing, and evaluating a KT strategy encouraged students to collaborate together to prepare a presentation on the final day of the institute. Faculty mentors acted as guides and facilitated the small group meetings to ensure that the students understood the task requirements. (Table 1 outlines the KTSI faculty, Table 2 summarizes the daily program and curriculum[6], and Appendix 2 outlines the small group project. Additional file 2 provides detailed information about the daily program and curriculum).

**Table 1:** Faculty members at the 2008 Canadian Institutes of Health Research Summer Institute

Name	Title(s)	Affiliation(s)
Laurie M. Anderson, PhD	Health Scientist	US Centres for Disease Control and Prevention

Richard Baker, MD	Professor of Quality Health Care Head, Department of Health Sciences	University of Leicester, United Kingdom

Melissa C. Brouwers, PhD	Associate Professor Provincial Director, Program in Evidence-based Care Project Lead, Capacity Enhancement Project	Department of Clinical Epidemiology and Biostatistics, McMaster University, Canada Cancer Care Ontario Canadian Partnership Against Cancer Corporation

Donna Ciliska, RN, PhD	Professor, School of Nursing Scientific Director	McMaster University, Canada National Collaborating Centre for Methods and Tools

Jill J. Francis, PhD	Health Psychology Lead, Health Services Research Unit	University of Aberdeen, United Kingdom

Ian D. Graham, PhD	Vice-President of Knowledge Translation	Canadian Institutes of Health Research

Jeremy M. Grimshaw, MD, PhD	Director, Clinical Epidemiology Program Canada Research Chair in Knowledge Transfer and Uptake	Ottawa Health Research Institute, Canada University of Ottawa, Canada

John N. Lavis, MD, PhD	Director and Investigator Canada Research Chair in Knowledge Transfer and Exchange	Program in Policy Decision-Making McMaster University, Canada

Doug G. Manuel	Senior Scientist Associate Professor	Institute of Clinical Evaluative Sciences, University of Toronto, Canada Department of Public Health Sciences, University of Toronto, Canada

Craig R. Ramsay	Programme Director Senior Statistician	Health Care Assessment Program of the Health Services Research Unit, Aberdeen, United Kingdom

Jon Salsberg, MA	Research Manager	Department of Family Medicine McGill University, Canada

Sharon E. Straus, MD, FRCPC, MSc	Associate Professor Canada Research Chair in Knowledge Translation	Department of Medicine, University of Calgary, Canada Department of Medicine, University of Toronto, Canada Li Ka Shing Knowledge Institute, University of Toronto, Canada

Charles Weijer, MD, PhD	Canada Research Chair in Bioethics	University of Western Ontario, Canada

**Table 2:** Summary of curriculum from the 2008 Canadian Institutes of Health Research Summer Institute

**Activity**	**Presenter**	**Topic**
**Day 1**		

Welcome	Jeremy Grimshaw	
Plenary	Ian D. Graham	Knowledge translation at CIHR
Plenary	Laurie M. Anderson	Knowledge for knowledge translation
Plenary	John N. Lavis	Knowledge translation for policy makers
In the spotlight	Ian D. Graham	Overview of his academic and career path from graduate school to current professional position.

**Day 2**		

Plenary	Jon Salsberg	Integrated knowledge translation (IKT)
Introduction to group work	Jeremy Grimshaw	
Group work		
KT in Action	Melissa C. Brouwers	Advancing the quality of cancer care: An intersection between KT/KTE research, a Health Service, and a Healthcare System
Plenary	Sharon E. Straus	Knowledge translation targeting healthcare professionals
Plenary	Jill Francis	Behavioural approaches to knowledge translation
Group work		
Plenary	Jill Francis and Jeremy Grimshaw	Developing knowledge translation interventions
Discussion/Group task	Sharon E. Straus	Mentorship[6]

**Day 3**		

Plenary	Jeremy Grimshaw	Knowledge translation research
Group work		
KT in Action	Doug Manuel	KT in action: Population benefit of Canadian Lipid Guidelines
Plenary	Craig Ramsay	Evaluating knowledge translation interventions
Group work		
Plenary	Donna Ciliska	Knowledge translation in public health
Plenary	Richard Baker	United Kingdom perspectives
Faculty and student interaction		Trainees had opportunities to book 15-minute one-on-one meetings with faculty members of their choice to discuss career plans or research.

**Day 4**		

Plenary	Charles Weijer	Ethics of knowledge translation and knowledge translation research
Group presentations	Trainees	

Among trainees, there was a sense that the mix of different learning forums informed by educational theories about adult learning factored greatly into the success of the KTSI. For example, didactic lectures from faculty, one-on-one meetings between trainees and faculty, and active learning sessions where we worked through a 'real' KT problem in small groups enabled an effective learning environment. From our perspective, the small group work provided the most useful opportunity to apply our new and existing knowledge of KT because it gave us time and space to interact with our peers and to learn by doing. Thus, we had the freedom to learn as we worked, the chance to turn to faculty mentors when we needed them, and the opportunity to see first-hand the complexity, confusion, and multiple stages required in developing a KT strategy.

In our small group task, we developed a KT strategy to reduce inappropriate antibiotic use in primary care (Appendix 2, task 5; Additional file 3). As a diverse multidisciplinary group, we struggled with our different (and sometimes conflicting) perspectives, which varied from perceptions of healthcare terminology (e.g. definition of primary care) to different conceptual approaches to problem solving (e.g. use of logic models). Our facilitators helped us constructively negotiate our differences by enabling group synergy, reinforcing trust and respect among team members, and creating a safe space for diverse voices. We found that working through the task was an important part of experiencing how to carry out KT research. Thus, our group work informs our lessons presented herein. Additional file 4 outlines our slide deck from our final presentation.

#### Key lessons learned

Because the KTSI provided us with many diverse opportunities to learn and share knowledge, we all continuously drew our own lessons and ideas. However, there were some key lessons that resonated within our small group. We share these lessons here because we think they highlight the essence of our experience and demonstrate how education and training can facilitate a deeper understanding and passion for KT. Our discussion will also highlight the implications of these lessons for future training initiatives.

#### KT is interdisciplinary and collaborative

Because the goal of KT is to use research in healthcare practice, it inherently involves partnership. Therefore, researchers from various disciplines (e.g. sociology, medicine, psychology, nursing, nutrition, engineering) engage in KT research, and we need different people to fill many roles within the context of the research. The CIHR distinguishes between end-of-grant KT and integrated KT (IKT) [3,7]. In the former case, this partnership may extend beyond the core research team at the end of the project to include communications experts to help with the dissemination of findings. In the latter, partners are engaged throughout the research process, from the development of the research question to its dissemination. Thus, IKT is often likened to participatory action research (PAR), which includes similar principles of engagement, partnership, and reciprocity in research [8,9].

### Negotiation skills are integral

We learned that because KT is interactive and collaborative, good negotiation skills and an effective mediation strategy are necessary to keep a large-scale research project, including its multiple researchers, partners, and support staff, on track. Through our group work, we identified the importance of negotiation and found that even in this brief time, creating a safe space to allow team members to express ideas, and finding ways to manage our differences in opinions and perspectives were keys to our success. We appreciated our assigned faculty members who acted as facilitators and content experts.

### The KT process is complex, confusing, and multifaceted

The plenary sessions, and particularly our small group work, taught us that having negotiation strategies and supports are essential in the 'real world' of KT. While this means that KT research is often 'messy', it also means that it is interesting, engaging, and can be an incredible learning experience for the research team. For example, although the small group work was complex and frustrating at times, we ultimately connected as a team, learned a lot about ourselves and about each other, and gained valuable real-world experience.

### Use the most rigorous methods of inquiry to answer different research questions

Although most of the research presentations at the KTSI focused on quantitative methods, participants expressed interest in hearing about research utilizing qualitative and/or mixed methods to understand and evaluate KT. We were reminded at the KTSI to be cautious not to fall into an 'us versus them' (*i.e*., qualitative versus quantitative methodologies) quagmire in doing KT research, but instead to foster interdisciplinary research and evaluation in addition to ensuring interdisciplinary care provision in healthcare.

The lessons described above exemplify the breadth and depth of the information gathered by participants at the KTSI. We received a sound understanding of the theory and practice of KT and had a healthy discussion about the benefits of qualitative and quantitative methods. We believe, however, that the overall success of the Institute was due to the adult-centered education techniques and opportunities to actively apply our knowledge in the small group project. Opportunities like the KTSI, and the lessons they provide trainees are truly enriching and will have a long-lasting effect on the discipline of KT.

### Riding the KT roundabout: reflections on the KTSI

For our group, Dr. Melissa Brouwers's presentation and her metaphor of a traffic roundabout helped us make sense of the lessons we learned and experiences we had at the KTSI. As Dr. Brouwers explained, in KT, the continuous stream of traffic around the central island represents the core research team in a KT project: this group has a constant presence and is engaged throughout the project. The vehicles entering in and out of the roundabout represent the various partners and stakeholders (e.g. community members, content experts, service delivery personnel, methodological experts, policy makers, users, evaluators) who provide input and expertise along the way. Engaging people at the right time and the right place is essential for ensuring that there are no KT accidents!

While the roundabout metaphor presented by Dr. Brouwers was useful for understanding the process of KT research, we also found that it spoke to our group's experiences at the KTSI. In essence, we, the participants, are the next generation of KT researchers, and the KTSI taught us the initial 'rules of the road'. For instance, the activities of the institute helped us learn how to negotiate the complexities of the field and understand its multiple dimensions. Both formal and informal mentorship provided by the faculty supported and encouraged us to chart a path of our own, learn from our own mistakes, and reach our own conclusions. By way of modeling and actively engaging in mentorship, the KTSI faculty members helped trainees realize how and when to utilize each other's strengths to overcome our individual and collective weaknesses.

### KTSI workshop outcomes

The KTSI facilitated many invaluable opportunities for its participants, and we suggest this model may be helpful to inform future training initiatives internationally. The KTSI formed an international network of participants with interests in KT and facilitated important interpersonal relationships between trainees and faculty. All attendees expressed interest in maintaining relationships, keeping abreast of each other's work, and participating in future KT training opportunities. Post-KTSI, the faculty initiated the development of an electronic mailing list and website informing participants of upcoming international KT opportunities for training and funding . This paper is just one example of the many outcomes that have arisen from the KTSI's network and faculty-trainee mentorship relationships. In another example, electronic communication between KTSI participants and faculty helped inform the curriculum for a conference workshop on KT; one participant secured a job following the KTSI. The variety of outcomes from the KTSI (e.g. newly formed relationships, sharing of ideas and resources, active scholarship) are a testament to the success of the workshop.

### Strengths and limitations of the KTSI

Participant feedback identified the following strengths of the workshop: the breadth and variety of workshop content, enthusiasm of faculty members, opportunities to interact with faculty members, and career planning and mentorship discussions. Suggestions for improvement included allowing more time for informal discussions and networking among participants and faculty, more discussion on use of qualitative methods and health economics in KT, and discussions of additional applications of KT in other aspects of health (e.g. organizational, social, educational).

From our perspective, key strengths of the KTSI included the interdisciplinary backgrounds of the participants, use of adult-centered educational learning techniques, and opportunities for active learning through small group projects. Suggestions for improvement include providing more information on the complementary nature of qualitative and quantitative methods, more opportunities to interact with faculty, and more detailed discussion of career options. We suggest that considerations for future initiatives include facilitating ongoing communication between participants and faculty, and offering future opportunities for in-person interactions between participants and faculty.

## Conclusion

We take away from our first traffic lesson provided at the KTSI insight about the importance of relationships, the complexity of interactions, the significance of timing, and the potential for ingenuity and innovation in the field of KT. These lessons are important for us as we strive to situate ourselves within the field of KT research, and for others interested in and/or already engaged in the field. Because of our positive experiences at the KTSI and the proven benefits of mentorship and training, we advocate for a continued focus on the next generation of KT researchers. We encourage other national and international KT organizations and funding agencies to consider replicating the training model employed here, as investment into KT personnel will foster the advancement of the field within and beyond local borders.

## Competing interests

The CIHR funded the authors' travel and accommodation at the Summer Institute, and paid for the article processing charge. Michelle Kho is funded by a Fellowship Award through the CIHR (Clinical Research Initiative).

## Authors' contributions

MEK conceived the design. MEK and EAE lead the coordination and integration of author comments and response to reviewers. All authors contributed to data acquisition, analysis, and interpretation of the data. All authors were involved in drafting the manuscript, critical revisions for important intellectual content, and gave final approval of the version to be published.

## Authors' information

MEK is a registered physical therapist and a PhD candidate. EAE is currently working as a research coordinator involved in research focused on KT, diabetes care, and Aboriginal health. RTD is a doctoral student in the field of Health Promotion. LM is a currently a clinical psychology intern and a PhD candidate in Clinical Psychology. BLB recently completed her PhD and is currently working as a Research Coordinator.

## Appendix 1: Applicant questions

1. Write a brief description describing your current research project or plans, and how KT and/or KT research is embedded within them (maximum 300 words).

2. Write a brief description of your expectations of the Summer Institute on Knowledge Translation and Knowledge Translation Research and how the Summer Institute experience fits with the direction of your studies or career path (maximum 500 words).

3. Please outline here any voluntary, work, or practice experience that you have that would be relevant for understanding why you wish to attend our Summer Institute and the experience that you bring with you (maximum of 500 words).

## Appendix 2: Small group task

1. Tasks

1. Design a KT strategy for CHSRF Evidence Boost – Allow midwives to participate as full members of the healthcare team.

2. Design a KT strategy for CHSRF Mythbusters – The risks of immunizing children often outweigh the benefits.

3. Design a KT strategy for CHSRF Mythbusters – Direct-to-consumer advertising is educational for patients.

4. Design a KT strategy for the Capacity Enhancement Program of the Cancer Guidelines Action Group of the Canadian Partnership Against Cancer Corporation.

5. Design a KT strategy to reduce inappropriate use of antibiotics for upper respiratory tract infections in primary care settings.

2. Design and evaluation considerations

1. What should be transferred? To whom should research knowledge be transferred? With what effect should research knowledge be transferred?

2. What are the likely determinants (barriers and facilitators) of KT?

3. By whom should research knowledge be transferred? How should research knowledge be transferred?

4. How will you know whether the KT strategy was effective? How will you know why your KT strategy was/was not effective?

Legend for Appendix 2: In this appendix, we outline the five different KT challenges taken on in the small group work as well as the design and evaluation considerations for the small group tasks. CHSRF: Canadian Health Services Research Foundation.

## Supplementary Material

Additional file 1 **Authors' research and relationship to KT and/or KT research (Essay question one)**. Authors' responses to essay question 1, 'Write a brief description describing your current research project or plans, and how KT and/or KT research is embedded within them.' Click here for file

Additional file 2 **Detailed curriculum from the 2008 Canadian Institutes of Health Research Summer Institute**. Additional information complementary to Table 2. Description of each presenter's talk. Click here for file

Additional file 3 **Sample small group task**. Group five small group KT task Click here for file

Additional file 4 **Reducing inappropriate antibiotic use in primary care: developing a KT strategy**. Final slide deck from authors' small group task at the CIHR Summer Institute Click here for file
