# Supplementary material for: Riding the knowledge translation roundabout: lessons learned from the Canadian Institutes of Health Research Summer Institute in knowledge translation
Source: Implement Sci. 2009 Jun 12;4:33. doi: 10.1186/1748-5908-4-33 (PMC2700786; doi:10.1186/1748-5908-4-33)
Supplement: Additional file 1 — Authors' research and relationship to KT and/or KT research (Essay question one). Authors' responses to essay question 1, 'Write a brief description describing your current research project or plans, and how KT and/or KT research is embedded within them.' [file 1748-5908-4-33-S1.pdf]

Additional file 1: Authors' research and relationship to KT and/or KT research (Essay question 1)

Author	Write a brief description describing your current research project or plans, and how KT and/or KT research is embedded within them.
Bell	Knowledge translation issues have become important in my current studies and work. I am completing my doctoral dissertation on Canadian youth as participatory citizens and my present employment as a researcher with the CIHR-funded Atlantic Networks for Prevention Research (ANPR), presents a strong emphasis on KT issues.<br>Concerning my own research, I am eager to better understand how to translate my findings beyond an academic environment in order to influence youth-focused policy and community-level change. My doctoral research and thesis writing have helped me to develop a number of important skills; however, KT beyond academia is rarely addressed. I welcome the opportunity to develop my understanding of KT in order to apply it effectively in the future development of my own research, perhaps as a post-doctoral fellow.<br>In my capacity as a researcher with the ANPR, KT is at the core of the project goals, particularly developing ways to facilitate meaningful communication between the stakeholders involved at the micro and macro levels of school health: students, parents, teachers, administrators, community organizations, and policy makers. It is a challenge to effectively communicate research goals and findings among these groups, particularly considering differing language capacity, diverse backgrounds, and existing inequalities. In addition, KT forms the basis of successful collaboration with these groups towards developing and implementing participatory research projects. Sound KT research is fundamental to the health of communities, schools, and youth yet there are many gaps in the understanding and development of means for collaboratively working towards school health goals in Atlantic Canadian communities.
DeForge	I plan to examine and further develop the Promoting Action on Research Implementation in Health Services (PARHIS) framework developed by Kitson and colleagues (1998, 2008), whereby the Successful Implementation of change is deemed a function of the Evidence being introduced, the Context into which it is being introduced, and the way in which change is Facilitated.<br>Such an endeavor will be underpinned by a critical realist philosophy. I mention this as it has important ramifications in terms of how Evidence is conceived. Specifically, "evidence" will be considered along a continuum rather than a hierarchy, affording equal privilege to quantitative, qualitative, and experiential knowledge. This is consistent with a critical realist approach to inquiry in that it attends to ontological, epistemological, practical, as well as relational dialectical dialogue.<br>Methodologically, critical ethnography will be layered upon my philosophical underpinnings as a means of examining the values, beliefs and behaviours that exist within the healthcare setting I engage with. In doing so, I and my participants will sensitize ourselves to the Contextual nuances of the setting, particularly with regards to where power (disparity) lies, which will be essential to Successful Implementation.<br>My Facilitation strategy will draw on theories of social change and of health promotion, with a particular focus on empowerment. This too remains consistent with critical realism as I seek to provide space and/or voice for that which is (or those who are) key mechanism(s) for triggering desired changes.<br>Finally, as a means of both Facilitating change and measuring Successful Implementation, I will adapt an existing goal attainment metric to my research, affording me a tool that: enables users to individualize goals within a specific setting; individualizes the scale on which attainment is measured; accommodates

Author	Write a brief description describing your current research project or plans, and how KT and/or KT research is embedded within them.
	multiple goals; and permits calculation of an overall score than enables comparisons between and within settings.
Estey	Exposure to documentation of the disproportionate burden of ill health faced by Aboriginal Peoples in Canada through my academic and work experiences has raised questions for me about the gap between what is known and what action is being taken to improve Aboriginal health in Canada. I have most actively explored this puzzle of knowledge translation (KT) through my Interdisciplinary (by special arrangement) Master's degree at the University of Victoria (UVIC). My thesis was grounded by a conceptual framework, which synthesized the KT literature with the Aboriginal health research literature. Using this framework as a guide, this study explored the idea of KT within one Aboriginal health research context – the Network Environments for Aboriginal Research British Columbia (NEARBC). Concepts, ideas, and patterns drawn from the systematic thematic analysis of semi-structured qualitative interviews highlighted the complexity of Aboriginal KT and the challenges that lie ahead.<br>In order to build on the lessons learned from this work and further engage with the topic of Aboriginal KT, I will continue my research as a Doctoral student in the Political Science department at UVIC in September. The broad research question driving my PhD will be: "what are the theoretical and practical components of knowledge translation in Aboriginal health?" Through qualitative research methods, such as semi-structured interviews and open-ended questionnaires, researchers, Aboriginal communities, policy-makers, and practitioners will be asked to comment on what knowledge they think should be translated, why this knowledge should be translated, and how this knowledge should be translated. While seemingly straightforward, these questions touch on deep philosophical issues related to KT, such as the classification of knowledge, the purpose of knowledge, and the reasons why knowledge should be transformed into action.
Kho	Can Knowledge Translation (KT) happen too soon? The evolution of an evidentiary base of new healthcare technologies (HT) often unfolds with contradictory information of variable quantity and quality. However, policy decisions, health care resource allocation and implementation in clinical settings often occur before the evidence matures with definitive results. Consider trastuzumab (Herceptin) for early stage breast cancer. Initial data were only publicly available as a conference presentation, and, based on these data alone, patients and clinicians immediately demanded access to the HT. Canadian policymakers, faced with incomplete information, were forced to make a funding decision, balancing promising initial results, no survival data, cardiac side effects, and tremendous costs. With an aging population and limited resources, the Canadian healthcare system is vulnerable to making HT decisions in this suboptimal manner. The purpose of my research program will address these limitations by developing, applying, evaluating and refining 2 new methodological approaches to evaluate new HTs, specifically: 1. To examine the completeness of reporting and natural history of scientific abstracts; and 2. To characterize the quantity, quality, and consistency of data through a new methodology named the Systematic Bibliometric Review (SBR).<br>My research program will utilize new methodologies and tools to objectively examine information on new HTs. Scientific abstracts will be critically assessed. The proposed new SBR methodology will describe the chronology of benefit and harm disclosure, incorporate quality assessment and temporal analysis to examine the susceptibility of citations to bias and change over time, and provide a comprehensive profile of an emerging technology, including initial, non-randomized data. Rituximab for non-Hodgkin's lymphoma is a compelling example to

Author	Write a brief description describing your current research project or plans, and how KT and/or KT research is embedded within them.
	demonstrate the value of these combined approaches for patients, clinicians, researchers, and policy makers.
Mak	My research focuses on knowledge translation by examining parents' desire for involvement in decision making concerning treatment, what information they wish to have concerning various treatments for child anxiety, what information is important in their decision making, and how they wish to receive the information. Our team will use the findings of this research to develop knowledge translation information for parents of anxious children and for clinicians providing information to parents. One type of knowledge translation product will be decision aids for parents to facilitate informed decision-making.
