# Supplementary material for: Riding the knowledge translation roundabout: lessons learned from the Canadian Institutes of Health Research Summer Institute in knowledge translation
Source: Implement Sci. 2009 Jun 12;4:33. doi: 10.1186/1748-5908-4-33 (PMC2700786; doi:10.1186/1748-5908-4-33)
Supplement: Additional file 2 — Detailed curriculum from the 2008 Canadian Institutes of Health Research Summer Institute. Additional information complementary to Table 2. Description of each presenter's talk. [file 1748-5908-4-33-S2.pdf]

Additional file 2: Detailed curriculum from the 2008 Canadian Institutes of Health Research Summer Institute

Activity	Presenter	Topic
**Day 1**		
Welcome	Jeremy Grimshaw	
Plenary	Ian D. Graham	Knowledge translation at CIHR<br>• CIHR's conceptualization of KT<br>• Presentation of CIHR's definition of KT (integrated and end of grant) and CIHR's KT strategy[1]<br>• Overview of CIHR's KT funding opportunities
Plenary	Laurie M. Anderson	Knowledge for knowledge translation<br>• Overview of the challenges faced in utilizing systematic reviews for policy-making purposes
Plenary	John N. Lavis	Knowledge translation for policy makers<br>• Presentation on KT in policy contexts, and the similarities and differences between policy and clinical KT practices
In the spotlight	Ian D. Graham	Overview of his academic and career path from graduate school to current professional position.
**Day 2**		
Plenary	Jon Salsberg	Integrated knowledge translation (IKT)<br>• Overview of IKT in contrast to end-of-grant KT: Emphasis of IKT on

Activity	Presenter	Topic
		researcher-participant collaboration throughout the entire research process[2]
Introduction to group work	Jeremy Grimshaw	
Group work		
KT in Action	Melissa C. Brouwers	Advancing the quality of cancer care: An intersection between KT/KTE research, a Health Service, and a Health Care System<br>• Knowledge translation described as a traffic roundabout and the need to carefully engage different stakeholders at the right time, optimizing their expertise.<br>• Discussion of the role of knowledge transfer techniques in helping health care providers, administrators, and government make better decisions.<br>• Description of Cancer Care Ontario's Program in Evidence-Based Care evidence-based advice incorporating stakeholders' perspectives in evidence production and review.
Plenary	Sharon E. Straus	Knowledge translation targeting health care professionals<br>• Outline of different means of assessing practitioner needs, emphasizing the importance of local context<br>• Discussion of barriers to knowledge-to-action (e.g., time, skepticism, accessibility of evidence, etc.)<br>• Overview of strategies for assessing and evaluating KT interventions
Plenary	Jill Francis	Behavioural approaches to knowledge translation

Activity	Presenter	Topic
		• Consideration of the usefulness and application of theories (i.e., theory of planned behaviour) in shaping a KT strategy.[3]<br>• Discussion of some factors involved in behaviour change when considering the effects of a KT intervention.
Group work		
Plenary	Jill Francis and<br>Jeremy Grimshaw	Developing knowledge translation interventions<br>• Discussion of behaviourally-focused approach to informing knowledge translation interventions<br>• Use of intervention mapping and matching intervention techniques to theoretical constructs to strategically design studies[4]
Discussion / Group task	Sharon E. Straus	Mentorship[5]
**Day 3**		
Plenary	Jeremy Grimshaw	Knowledge translation research<br>• Overview of the inherently interdisciplinary nature of KT research as a relatively new and broad field<br>• Current evidence base provides little practical guidance for health care systems about which interventions are best<br>• An emerging body of evidence shows that it is possible to change stakeholder decisions and behaviours
Group work		

Activity	Presenter	Topic
KT in Action	Doug Manuel	KT in action: Population benefit of Canadian Lipid Guidelines<br>• Overview of a data-driven approach to estimate the population impact of implementing Canadian lipid guidelines[6]<br>• Describes *synthesis, partnerships, & evaluation* as three fundamentally important components of successful KT
Plenary	Craig Ramsay	Evaluating knowledge translation interventions<br>• Cluster Randomized Trials (RCT) as the 'gold standard' for evaluating KT<br>• Advantages of using RCT: evidence that the effects of the KT intervention are attributable to the intervention, i.e., higher internal validity. Challenges of using RCT: must be cautious of inter-cluster effects; need large sample sizes<br>• Interrupted Time Series design an alternative if a RCT is not possible; this mitigates some of the error inherent in simple pre-post designs
Group work		
Plenary	Donna Ciliska	Knowledge translation in public health<br>• Overview of the National Collaborating Centre for Methods and Tools, which focuses on methods and tools for knowledge synthesis, translation, and exchange<br>• The Center conducts KT in public health, evaluation studies, and capacity development and provides tools such as PublicHealth+, health-evidence.ca, etc.<br>• Outlined one particular study on the use of a knowledge broker in moving evidence into practice for obesity prevention

Activity	Presenter	Topic
Plenary	Richard Baker	United Kingdom perspectives<br>• Provided an overview of the national improvement program and pay for performance scheme in the UK<br>• Described the impact of the 1997 election of the New Labour Party in the UK, thus providing a historical context to the progress of KT in the UK
Faculty and student interaction		Trainees had opportunities to book 15-minute one-on-one meetings with faculty members of their choice to discuss career plans or research.
**Day 4**		
Plenary	Charles Weijer	Ethics of knowledge translation and knowledge translation research<br>• Provided an overview of research ethics and implications for knowledge translation research<br>• Initiated a healthy discussion of differences between quality assurance initiatives and research using a current exemplar
Group presentations	Trainees	

References

1. **Knowledge translation strategy 2004-2009** [http://www.cihr-irsc.gc.ca/e/26574.html]
2. Salsberg J, Louttit, S., McComber, A.M., Fiddle,r R., Naqshbandi, M., Receveur, O., Harris, S.B., Macaulay, A.C.: **Knowledge, Capacity and Readiness: Translating Successful Experiences in CBPR for Health Promotion**. *Pimatisiwin: A Journal of Indigenous and Aboriginal Community Health* 2008, **5**:125-150.
3. Michie S, Johnston M, Abraham C, Lawton R, Parker D, Walker A: **Making psychological theory useful for implementing evidence based practice: a consensus approach**. *Qual Saf Health Care* 2005, **14**:26-33.

4. Abraham C, Michie S: **A taxonomy of behavior change techniques used in interventions**. *Health Psychol* 2008, **27**:379-387.
5. Sackett DL: **On the determinants of academic success as a clinician-scientist**. *Clin Invest Med* 2001, **24**:94-100.
6. Manuel DG, Tanuseputro P, Mustard CA, Schultz SE, Anderson GM, Ardal S, Alter DA, Laupacis A: **The 2003 Canadian recommendations for dyslipidemia management: revisions are needed**. *CMAJ* 2005, **172**:1027-1031.
