# Supplementary material for: Riding the knowledge translation roundabout: lessons learned from the Canadian Institutes of Health Research Summer Institute in knowledge translation
Source: Implement Sci. 2009 Jun 12;4:33. doi: 10.1186/1748-5908-4-33 (PMC2700786; doi:10.1186/1748-5908-4-33)
Supplement: Additional file 3 — Sample small group task. Group five small group KT task [file 1748-5908-4-33-S3.pdf]

Additional File 3: Sample small group task

Small Group 5

Design a knowledge translation strategy to reduce inappropriate use of antibiotics for upper respiratory tract infections in primary care settings

Background

Antibiotic resistance is one of the world's most pressing public health problems. There is compelling evidence that exposure of the community and the individual to antibiotics enhances the risk of an individual harbouring a resistant micro organisms. One approach to reducing the incidence of infections due to antibiotic-resistant organisms is to reduce the inappropriate use of antibiotics in both the hospital and community settings.

Symptoms of upper and lower RTIs are common presenting features in primary care and they are frequently treated with antibiotics. Over a 12 month period a full time family practitioner will have over 500 consultations for all RTIs. Antibiotic treatment offers little or no benefit to otherwise healthy patients in primary care settings and any benefits need to be weighed against possible adverse events and risk of community antibiotic resistance.[eg1;2] These findings have led the Centers for Disease Control and Prevention in the United States to recommend that antibiotics should not be prescribed for acute uncomplicated respiratory tract infections in healthy adult patients.[3-5] Reducing antibiotic prescribing in the community by the "prudent" use of antibiotics is seen as one way to slow the rise in antibiotic resistance and appears safe. However, our understanding of how best to achieve this is limited. One recent systematic review explored methods of improving antibiotic resistance in primary care finding 'Use of printed educational materials or audit and feedback alone resulted in no or only small changes in prescribing. The exception was a study documenting a sustained reduction in macrolide use in Finland following the publication of a warning against their use for group A streptococcal infections. Interactive educational meetings appeared to be more effective than didactic lectures. Educational outreach visits and physician reminders produced mixed results. Patient-based interventions, particularly the use of delayed prescriptions for infections for which antibiotics were not immediately indicated effectively reduced antibiotic use by patients and did not result in excess morbidity. Multi-faceted interventions combining physician, patient and public education in a variety of venues and formats were the most successful in reducing antibiotic prescribing for inappropriate indications'.[6]

Task

You are part of an Antibiotic Resistance Task Force for a regional health authority that is developing a knowledge translation strategy to reduce inappropriate use of antibiotics in primary care settings.

References

(1) Del Mar CB, Glasziou PP, Spinks AB. Antibiotics for sore throat. Cochrane Database Syst Rev 2006;(4):CD000023.

(2) Glasziou PP, Del Mar CB, Sanders SL, Hayem M. Antibiotics for acute otitis media in children. Cochrane Database Syst Rev 2004;(1):CD000219.

(3) Cooper RJ, Hoffman JR, Bartlett JG, Besser RE, Gonzales R, Hickner JM et al. Principles of appropriate antibiotic use for acute pharyngitis in adults: background. Ann Intern Med 2001; 134(6):509-517.

(4) Snow V, Mottur-Pilson C, Gonzales R. Principles of appropriate antibiotic use for treatment of acute bronchitis in adults. Ann Intern Med 2001; 134(6):518-520.

(5) Snow V, Mottur-Pilson C, Gonzales R. Principles of appropriate antibiotic use for treatment of nonspecific upper respiratory tract infections in adults. Ann Intern Med 2001; 134(6):487-489.

(6) Arnold SR, Straus SE. Interventions to improve antibiotic prescribing practices in ambulatory care. Cochrane Database Syst Rev 2005;(4):CD003539.
