# Supplementary material for: Riding the knowledge translation roundabout: lessons learned from the Canadian Institutes of Health Research Summer Institute in knowledge translation
Source: Implement Sci. 2009 Jun 12;4:33. doi: 10.1186/1748-5908-4-33 (PMC2700786; doi:10.1186/1748-5908-4-33)
Supplement: Additional file 4 — Reducing inappropriate antibiotic use in primary care: developing a KT strategy. Final slide deck from authors' small group task at the CIHR Summer Institute [file 1748-5908-4-33-S4.pdf]

Reducing inappropriate antibiotic use in primary care: developing a knowledge translation strategy

2008 CIHR Summer Institute

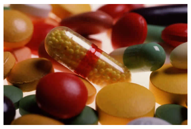

June 25, 2008

Brandi Bell
Ryan DeForge
Elizabeth Estey
Michelle Kho
Leanne Mak

CIHR IRSC

CIHR Institute of Health Services and Policy Research

Acknowledgements

- Donna Ciliska
- Jill Francis
- Sharon Strauss
- Andrea Smith
- Hannah Burleton
- Other observers
- CIHR Summer Institute

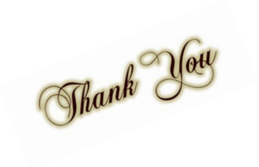

CIHR IRSC

CIHR Institute of Health Services and Policy Research

Context

- **Who**: Antibiotic Resistance Task Force – Regional Health Authority
- **What**: KT strategy to reduce the inappropriate use of antibiotics for URTIs in primary care settings
- **Why**: Evidence of individual and community risks associated with antibiotic resistance and use of antibiotics when they are non-essential

CIHR IRSC

CIHR Institute of Health Services and Policy Research

Outline

1. Description of multifaceted KT strategy/ intervention
2. Discussion of process and group reflections

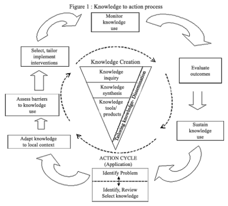

CIHR IRSC

CIHR Institute of Health Services and Policy Research

Getting started

- Logic Model approach
  - Phase 1: taskforce composition, stakeholder engagement
  - Inputs
  - Barriers/facilitators
  - Activities
  - Outputs
  - Outcomes

**Goals/ objectives**	**To decrease the prevalence of inappropriate prescriptions**
Inputs	• Developing the task force, integration of stakeholders
Barriers	• Time; current (habitual) practices
Facilitators	• Piggy-backing on existing opportunities; incentives (e.g. CMEs)
Activities	• Interactive educational meetings
Outputs	• Planned curriculum and materials
Outcomes	• Decrease in the number of inappropriate prescriptions

CIHR IRSC

CIHR Institute of Health Services and Policy Research

Overview of multifaceted KT intervention

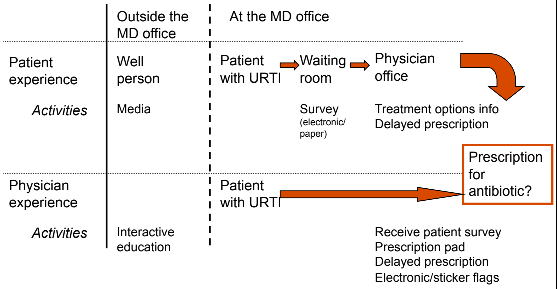

CIHR IRSC

CIHR Institute of Health Services and Policy Research

Evaluation Question

Does a multifaceted, community-based participatory KT strategy vs. no KT strategy aimed at family physicians reduce inappropriate antibiotic prescribing for upper respiratory tract infections in otherwise healthy adults and children?

CIHR IRSC

CIHR Institute of Health Services and Policy Research

Overview of pilot cluster randomized trial design

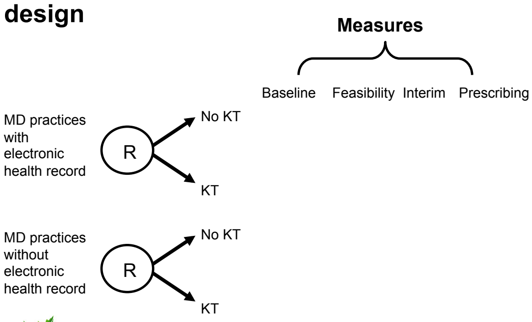

CIHR IRSC

CIHR Institute of Health Services and Policy Research

Process – Relationship Building

- Group enablers
  - Synergy
  - Trust and respect
  - Safe space
  - Diverse voices
- Facilitator role
  - Focusing
  - Prompting
  - Expertise
  - Tone
  - Summary

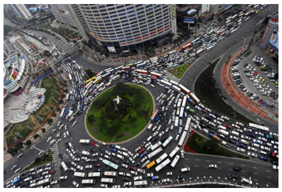

CIHR IRSC

CIHR Institute of Health Services and Policy Research

Process – Multidisciplinarity

- Clarifying language
  - “primary care setting”
  - “barriers”
- Negotiating and working with different models/approaches
  - Comfort levels varied
    - Created tension AND promoted creativity

CIHR IRSC

CIHR Institute of Health Services and Policy Research

What we’ve learned…

- Importance of negotiation skills
- A safe space is fundamental to collaboration
- Issues are complex and require time and resources to address fully
- Individuals fit into the KT process in different ways

CIHR IRSC

CIHR Institute of Health Services and Policy Research
